# Preliminary user centred evaluation of regional aircraft cabin interiors in virtual reality

**DOI:** 10.1038/s41598-021-89098-3

**Published:** 2021-05-06

**Authors:** F. De Crescenzio, S. Bagassi, F. Starita

**Affiliations:** 1 Department of Industrial Engineering, University of Bologna, Forlì, Italy; 2 Center for Studies and Research in Cognitive Neuroscience, Department of Psychology, University of Bologna, Cesena, Italy

**Keywords:** Psychology, Aerospace engineering

## Abstract

The main aim of the CASTLE (Cabin System Design Towards Passenger Wellbeing) European project is to deliver innovative interiors solutions that maximize the comfort and wellbeing of passengers in the next future. To achieve such objective, an effective HCD (Human Centred Design) approach has been employed to derive a Human Response Model based on a holistic assessment of comfort. The overall methodology has been conceived to provide different tools and methods to collect data on the impact that the design of each cabin item has on the user from the earliest design stages. One of these tools is represented by using 3D virtual mock-ups to capture data on the user’s perception and to rate the level of appreciation inspired by the specific design. In this paper we present the experimental procedures and the results from a preliminary experimental campaign of Human in the loop simulations in Virtual/Augmented Reality of a Regional Aircraft.

## Introduction

In a growing and interconnected commercial aviation system, being capable of offering to passengers a comfortable journey and of optimizing the time spent travelling makes companies more competitive and undoubtedly has a strong impact on travellers’ level of satisfaction/dissatisfaction. Usually, to improve the comfort in cabin interiors aircraft designers focused on the postural and/or on the noise and vibration (N&V) issues^1,2^. However, comfort recently started to be enlarged to a wider set of aspects, such as the general appreciation and visual comfort of passengers, as well as the perception of travelling in a comfortable cabin and the sensation of having enough space to work, have fun or rest during the journey. Several studies that have explored the concept of multisensory comfort and well-being in commercial and private aircrafts and are mostly collected in^3^. One context feature that is recognised as very much impacting the overall comfort of passengers is the legroom, or the space available for the knees while sitting, as well as the overall cabin appearance. With the current pandemic due to COVID-19 the personal space available in transport means assumes an even more important role in the total impact that it has on the passenger’s comfort. In fact, the perceived personal space can be related to the actual or perceived social distance from the other passengers and a wider perceived space may mean reduced anxiety of contagion.

This paper reports the results from the preliminary experimental campaign for the assessment of visual and interaction comfort in a regional aircraft virtual environment. The work has been conducted in the framework of a Research and Innovation project funded by the European Commission under the Horizon 2020 programme. The project title is CASTLE (Cabin Systems Design Toward Passenger Well-being), and it is part of the Clean Sky initiative^4^. Besides the main aim of increasing the competitiveness of the European aeronautic sector through the creation of cleaner aircraft and rotorcraft, this large European initiative started to foster an innovation process oriented to the improvement of the onboard passenger’s experience.

The present work also intends to validate the assessment procedure in terms of repeatability, easiness of conduction or under any other aspect that can prevent or favour the involvement of several participants and the update of the virtual environment with the latest revision of the design.

In the next sections, after a review of comfort and assessment methods in commercial aircraft interiors, the experimental procedure of the HCD of Regional Aircraft interiors implemented in CASTLE is described. The work done to create the Virtual Environment for different scenarios (User standing in the cabin, Galley, Lavatory) and the subjective evaluation of these cabin items is presented. Finally, results of the preliminary test campaign conducted in order to validate the process are also presented and discussed.

### Comfort and assessment methods in commercial aircraft interiors

In the recent past several researchers focused on improving passenger’s comfort in commercial aviation with the aim of identifying priorities, in terms of what mostly affects the wellbeing and what is most important for travelling people, and finding correlations between the interiors’ features and quality of travel. Vink et al. in^5^ highlight the subjective nature of comfort and that soft aspects—such as crew attention—also contribute to passengers’ comfort, together with physical characteristics of the aircraft—such as seat appearance. In^6^ the authors, in addition to an attempt to identifying an importance ranking in passenger’s comfort subjective perceptions, provide a structured passenger’s comfort experience model. Such model aims at creating a framework for studying the relationships between contextual features related to the experience of passengers and the perceived comfort as composed of physical, psychological, and physiological components. Among the comfort themes they also identify the proximity, as the personal space of individuals. A further model is proposed by Patel & D’Cruz, where also other factors, such as the pre flight environment, are included as contributing factors. They remark that each component of comfort is the effect of several different inputs and can also evolve during flights according to changing needs on the passenger’s side^7^. A recent study proposes an assessment method that includes the expectations of passengers in the satisfaction/dissatisfaction level of comfort achieved and classifies the impacting factors, such as noise in the cabin, physical indicators of the seat geometry, vibrations and many others into three levels: factors significantly affecting comfort, factors generally affecting comfort and factors slightly affecting comfort^8^. Such studies are mainly based on trip reports or on passengers’ interviews. On one side they provide an insight on the aspect mostly affecting the comfort, but on the other side this approach is based on existing solutions that cannot be changed or adapted to the user’s needs and/or comfort level and are hardly implemented in the actual design process. What if such creative process could be guided by final users by involving them in the creation of new cabin interiors far before the actual manufacturing of solutions? This is definitely possible thanks to the advancements gathered by Virtual Reality as a mean to immerse a potential user in a virtual environment of something not yet existing and to collect feedbacks on various aspects, such as ergonomics, usability or perceived comfort. The added value and the applicability of Virtual Reality in improved development processes have already been demonstrated in several application studies on interaction design^9^ and workplaces design^10^. An extensive study on how VR (Virtual Reality) can be implemented in vehicle design, and specifically in CAR industry, can be found in^11^. One of the cited topics regards the possibility to involve customers in evaluations in Virtual Mock ups.

Nevertheless, there are few examples of Virtual Reality used to involve aircraft passengers in commercial aviation. In^12^ the authors develop a method based on Virtual Reality to conduct human in the loop test for cabin accessibility. Moreover, Virtual Reality is used for conceptual evaluation of a private bubble for enhancing the sense of privacy in the travel experience of passengers^13^. Finally, in^14^ results from an European Project (VR-HYPERSACE) investigating the use of VR for in-flight use in cabins as a mean to improve comfort are presented.

The authors of this paper have used Virtual Reality for the validation of the conceptual design of a new generation business aircraft and for the comparison of different CMF (Colours and Material Finishing) proposals^15^.

In this paper a Human Response Model described in detail in^16^ is implemented. The method is mainly based on the definition of a repeatable methodology for the conduction of recurrent Human in the Loop validations. Such standardised assessment procedure is intended to collect the potential user’s feedback on the current state of the art of the design along the project development phases, lasting more than three years. Such phase starts from the early design to the PDR (Preliminary Design Review), to the CDR (Critical Design Review), the multi-disciplined technical review to ensure that a system can proceed into fabrication.

### Human in the loop simulation assessment—methodology for regional aircraft comfort evaluation in virtual reality

In the CASTLE Human in the Loop experiments, comfort metrics are evaluated in a virtual environment, in which a proper number and combination of voluntary subjects experience a virtual mock-up of one or more cabin items.

The general methodology follows the approach reported in^17^. It is composed of three steps: Experimental Planning, Experimental Execution and Data Collection, Data analysis and reporting. The Experimental Planning phase starts with collection of 3D CAD models to be uploaded in the Virtual Environment. In this paper we built the Virtual Environment starting from the CAD models of the seat, the lavatory, the galley, the cabin lining and the stowage bins.

Therefore, three different scenarios have been experimented. For each of the three scenarios a storyboard of tasks to be simulated in the virtual environment is designed to assess the comfort metrics of one or more cabin items replicated in the specific scenario.

**Figure 1:**
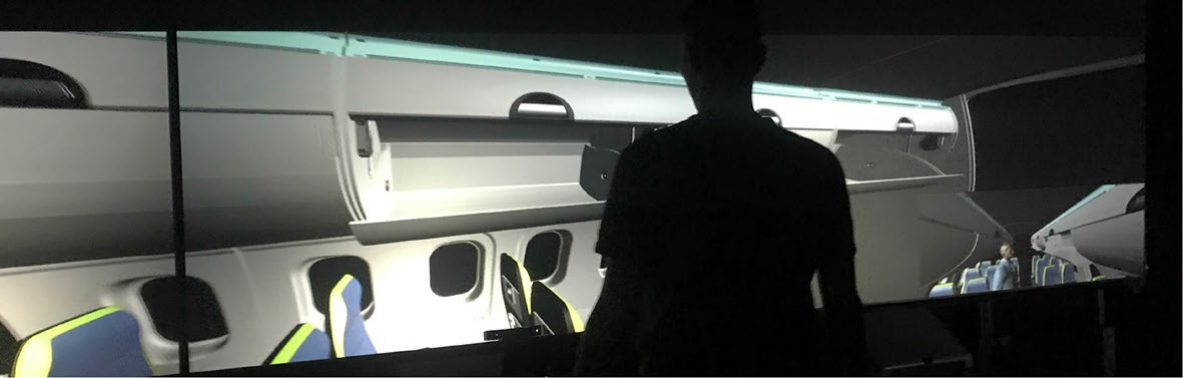
Participant in the CAVE.

The experiments took place at the Department of Industrial Engineering of the University of Bologna. One scenario is implemented in a CAVE (Cave Automatic Virtual Environment) and the other two are replicated in a See-Through Head Mounted Display. The CAVE is a multiple rear projected screens visualization system that immerses the user in a Virtual Environment. It was as invented by Cruz-Neira^18^. At the University of Bologna it is composed of three vertical screens measuring 2.5 × 1.9 m each and can be used in stereo mode wearing active stereoscopy glasses (Fig. 1). Each screen is rear projected through a BenQ W710ST8NVIDIA3D VisionReady DLP projectors. Resolution on each screen is 1.280 × 720 pixels. In the CAVE participants exploit the 1:1 scale visualization capability of a large model and enjoys hands free interaction. The participant is standing on the floor and his point of view and his joints are tracked by a Microsoft Kinect so that the egocentric view on the screen his updated with his actual viewpoint and an avatar replicates his movements^19^. In the right screen of the CAVE, at the lower bottom corner, an exocentric view of the avatar is placed in order to enhance the participant’s sensation of his position in the cabin. In the CAVE the participant interacts with the stowage bin door. If his avatar’s arm or his trunk collides with the door an audio and a visual signals are triggered as a feedback instead of the force feedback he would get in a real environment. The Micorsoft Hololens is a see-through display manufactured by Microsoft. In these experiments it is used in Mixed Reality mode. Hence, the participant experiences the single items, the galley and the lavatory, as if these were installed in the Laboratory. All the applications have been developed in Unity 3D.

Each participant is briefly introduced to the experiment and is asked to sign an informed consent declaration. Afterwards he/she is trained to the VR platform. After each scenario experiment, a questionnaire survey is submitted to the participant containing questions on the single items and regarding the different components of comfort, such as the visual comfort or the interaction comfort. Table 1 provides details of each phase and a description of the tasks to be performed and of the documents to be prepared.

**Table 1:** Description of the processes for the human in the loop experiments in VR.

CASTLE human in the loop assessment	
1st phase experimental planning	Item versus comfort metrics definition
	VR system set up
	Model type and features/configurations
	Storyboard description
	Questionnaire definition for subjective evaluation assessment
	Users to be involved/experimental total timing
2nd phase experimental execution and data collection (for each subject)	Recruitment and scheduling
	Informed consent signatory
	User training
	Run of the experiment and eventually collection of on line data
	Questionnaire provision to the user and debriefing
3rd phase analysis and reporting	Collection analysis of data coming from the experiments, in relation of simulation method and objective or subjective ranking
	Reporting

In this paper, we describe in detail the three scenarios as in Table 2. The right column of the table includes the model that have been imported in the corresponding scenario.

**Table 2:** Test case definition and connection with cabin items.

Description	Cabin items validated
Overall cabin assessment, with the user standing in the cabin (including navigation, seat row ingress/egress) and interacting with the stowage bin	Seat - stowage bin -lining
Galley assessment, with a user standing in front of it, exploring the model in real dimensions and interacting with it	Galley
Lavatory assessment, with the user standing inside it and exploring the model	Lavatory

### Overall cabin assessment, with the user standing in the cabin

In this scenario, the aim is to replicate the passengers experience during a specific phase of flight. In detail, it represents the phase in which the passenger approaches his/her seat and interacts with the stowage bin before taking his/her seat.

This scenario has been reconstructed in the CAVE and the virtual environment has been built upon the seat model, the stowage been model and the cabin lining model. Five seat rows and five stowage bins are reproduced in the scene. Some stow bins are open and some are closed. Such models have been arranged in the fuselage model so that an entire section of the cabin is created. At the time of the experiment, the surfaces of the cabin items were the actual surfaces produced in the framework of the CASTLE project while the colours were temporary since a separate colour study was still being conducted. The passenger is standing and can orient his/her sight to the seat row he is approaching and to the stowage bin.

**Figure 2:**
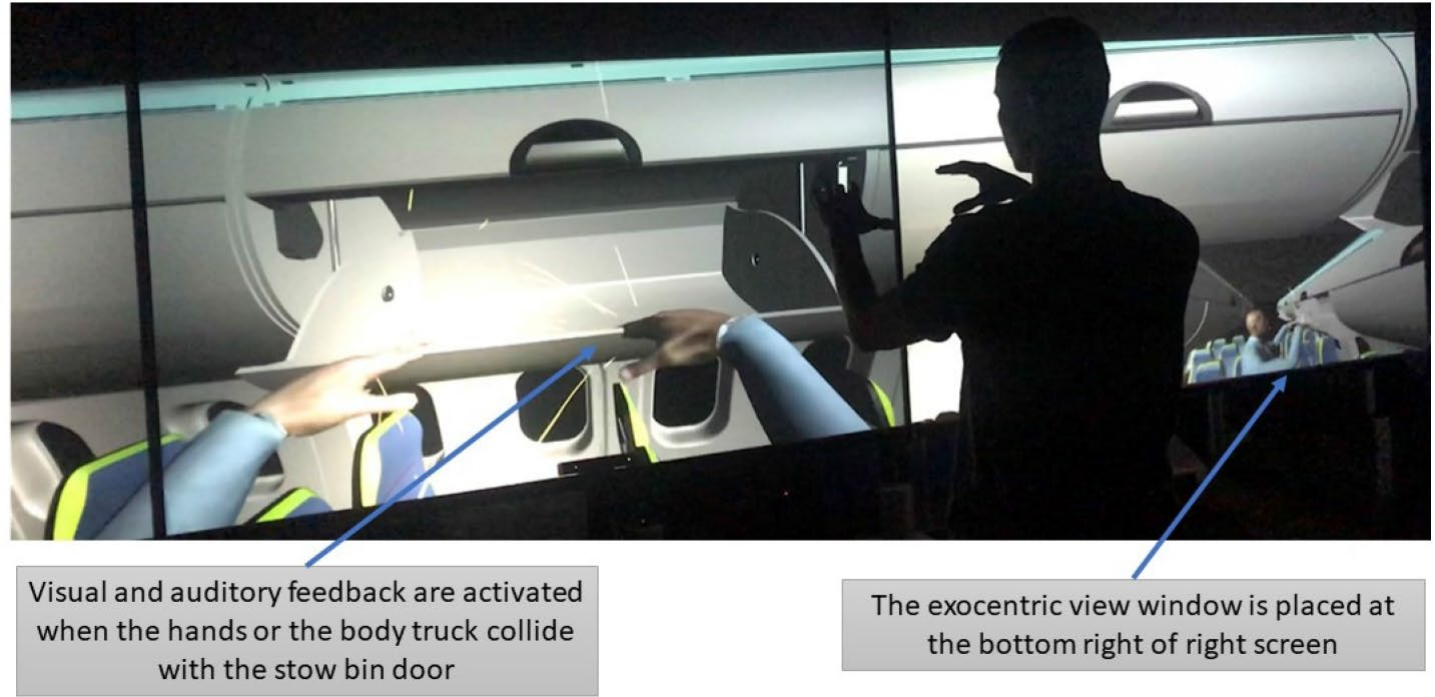
A participant interacting with the stowage bin.

Among the different components of comfort, in this campaign we aim to collect the general visual comfort, as the level of appreciation, the perceived living space comfort, as the level of living space that the passenger estimates at first sight, and the interaction comfort, as the extent to which he can interact with the cabin, knowing the limitations in space through the collision detection features. Collision detection is triggered by collisions between the avatar and the virtual cabin items, perceived by the passenger through auditory and visual feedbacks. As an example, the collision detection is activated for interferences between the avatar’s hands or forearms and the stowage bin doors as moving yellow lines originating in the collision area appear as the collision is detected (Fig. 2).

The questionnaire is based on a 5-point Likert scale where 1 = strongly disagree, 2 = mostly disagree, 3 = slightly agree, 4 = mostly agree, 5 = strongly agree and the questions for the general cabin scenario are related to the different cabin items that compose the scenario itself. Participants answer to the question: “Please, express how much you agree with each statement” for each of the statements in Table 3. Five-point Likert-type scale have been previously used to assess the subjective perception of cabin comfort^20–23^. Additionally, following^24^, we chose an asymmetric 5-point Likert scale (2 disagree, 3 agree), because it enables to have a more fine grained representation of the positive aspects in the design, while also giving the possibility to highlight the presence of any significantly critical features.

**Table 3:** Cabin scenario questionnaire.

Statement	Item
The seat seemed easy to access	Seat
The seat appeared to have enough space to stretch my legs	
I had the feeling of being in a spacious environment	
The space to access the seat and the leg room appeared sufficient	
I was pleased with the style/aesthetics of the cabin lining	Cabin lining
I had the feeling of well-being while walking in the cabin	
I was pleased with the style/aesthetics of the stowage bins	Stowage bin
I liked the shape design of the stowage bin	
The stowage bin appeared to be spacious enough to easily load my luggage	
The stowage bin seemed easy to reach/use	

### Galley assessment, with a user standing in front of the item

**Figure 3:**
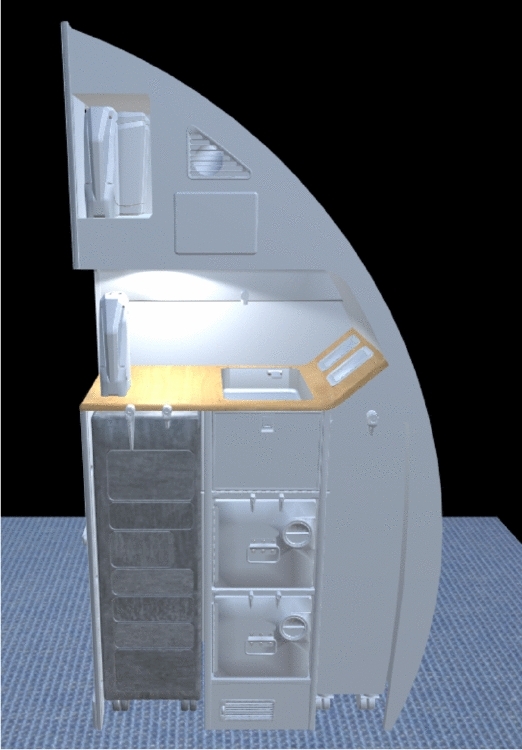
The virtual mock up of the galley.

In the Galley scenario a complete Virtual Model of the Galley has been derived from the CAD model. This model has been uploaded in a Microsoft Hololens. The user can explore the Galley and perceive the design in a 1:1 scale mock up projected in 3D (Fig. 3).

The components of comfort to be collected in this scenario are the visual comfort, as the level of appraisal, and the perceived interaction comfort as the user is asked to simulate interaction and rate if the objects seem easily reachable or cleanable.

Since this item is intended to be evaluated both by the passengers and by the cabin crew members the subjective evaluations strategy considers these two points of view. From a cabin crew point of view, the interaction is evaluated, to verify component reachability and cleanability. From a user/passenger perspective, the questionnaire is voted to evaluate design pleasantness and the style of the decorative finishing.

Again, participants answer to the question: “Please, express how much you agree with each statement” for each of the statements in Table 4 on a 5-point Likert scale where 1 = strongly disagree, 2 = mostly disagree, 3 = slightly agree, 4 = mostly agree, 5 = strongly agree.

**Table 4:** Galley scenario questionnaire.

Statement	Item
I was pleased with the style/aesthetics of the galley	Galley
The galley equipment appeared easy to reach	
The galley appeared easy to clean	

### Lavatory assessment, with the user standing in the lavatory

**Figure 4:**
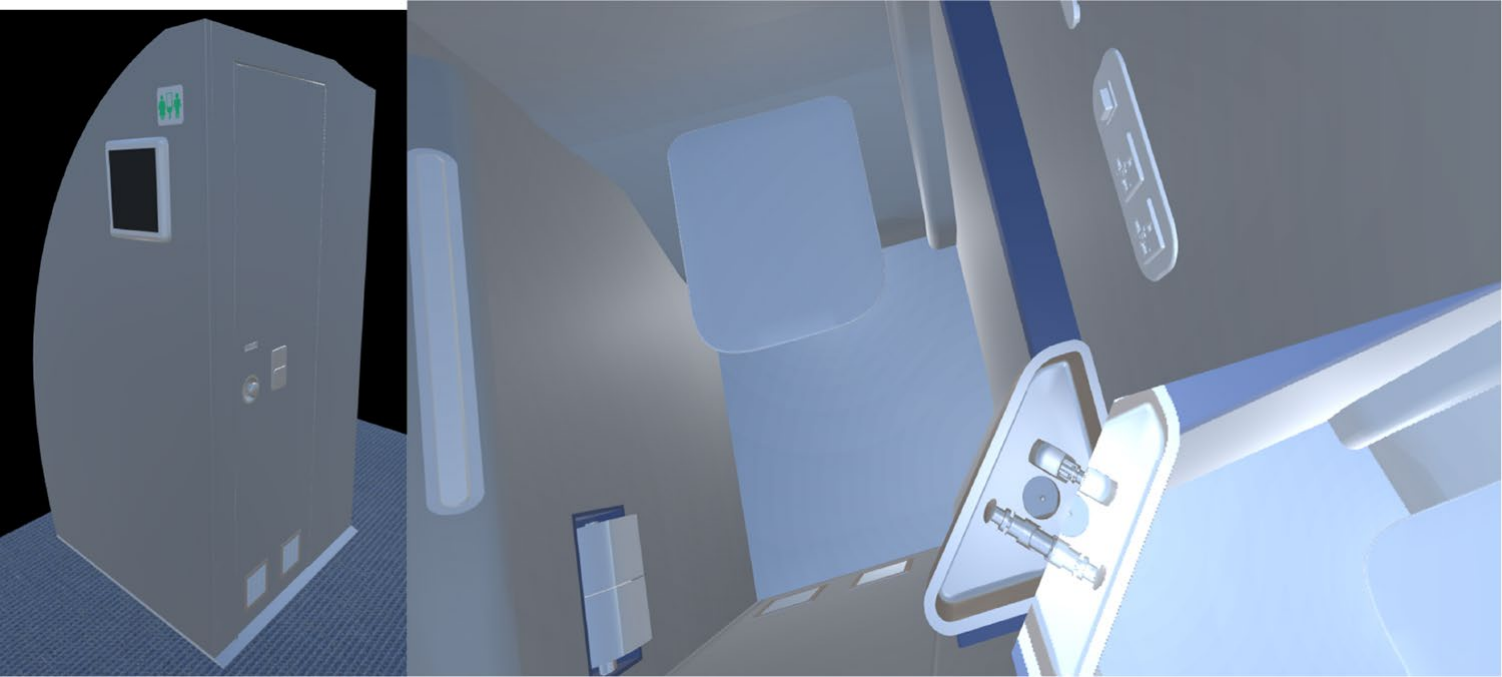
The virtual mock up of the lavatory.

In the lavatory scenario, the user also wears the Microsoft Hololens. He initially stands outside a 1:1 scale virtual mock-up of the lavatory, steps inside it through the door and explores the environment while standing in this narrow environment (Fig. 4).

The questionnaire aims to collect a visual feedback of lavatory accessibility and general aesthetic appearance. Questions are also aimed to predict some human factors aspects, such as the perception, through the immersion in the mock-up, of being able to operate with the different parts, such as the faucets.

Once more, participants answer to the question: “Please, express how much you agree with each statement” for each of the statements in Table 5 on a 5-point Likert scale where 1 = strongly disagree, 2 = mostly disagree, 3 = slightly agree, 4 = mostly agree, 5 = strongly agree.

**Table 5:** Lavatory scenario questionnaire.

Statement	Item
The lavatory appeared easy to access/exit	Lavatory
I was pleased with the style/aesthetics of the lavatory	
The lavatory appeared spacious	
The lavatory seemed easy to use	
The lavatory space seemed comfortable	

## Materials and methods

A preliminary test campaign has been conducted at the University of Bologna to gather a first set of data and to tune the experimental procedure.

### Participants

20 healthy subjects (16 males; age: M = 23 years, max = 30, min = 19) have been recruited among the University students. The study was approved by the Ethical Committee of the University of Bologna (file number: 187339, year: 2018) and all participants gave written informed consent before the beginning of the experiment. In addition to gender and age of each participant, the height (M = 174.85; SD = 6.2 cm) has also been recorded. This value has been used in the cabin environment to align the height of the avatar to the height of the participant in each session. The mean value is 174.85 cm and the all research was performed in accordance with relevant guidelines. All data were analyzed and reported anonymously.

### Procedure

**Figure 5:**
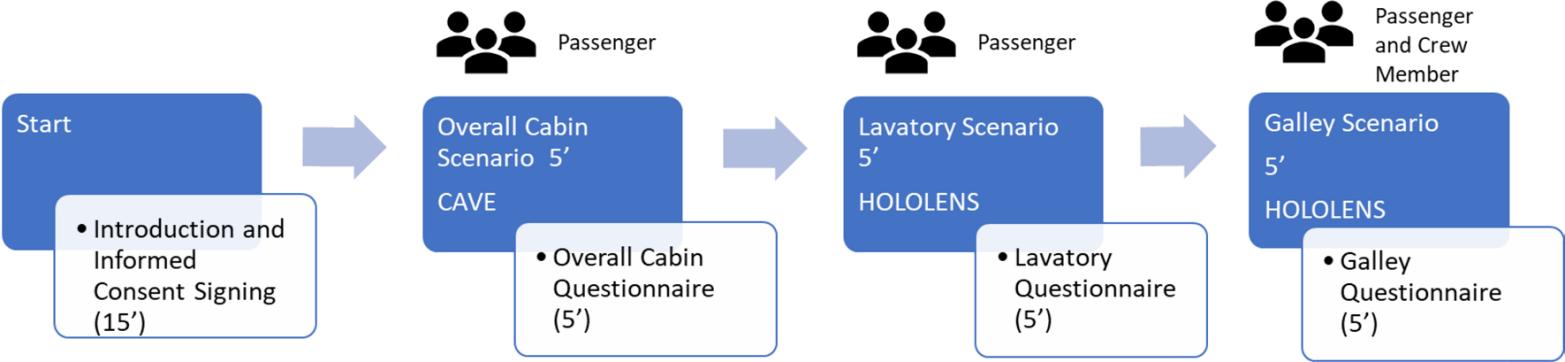
Procedure of the preliminary test campaign on the CASTLE regional jet.

Figure 5 shows an illustration of the experimental procedure. After, a brief introduction and the signing of the informed consent, the cabin scenario is started in the CAVE. Each participant is trained on the scenario basic aspects and on how to navigate in and interact with it before personally taking part to the experiment. Before starting, the height of the participant is inserted in the system so that the height of the avatar, and therefore of the virtual camera tracked with the Kinect, corresponds to participants’ height. Once the first simulation on the overall cabin scenario is completed, participants fill in the questionnaire concerning the seat and the stowage bin. Then, the participant wears the Hololens and, after a brief training on the system functioning, he experiences the virtual lavatory first and then the galley. Participants fill in the corresponding questionnaire after completing each scenario and before proceeding to the next one. The entire process lasts about 45’.

### Ethical approval

The study was approved by the Ethical Committee of the University of Bologna (file number: 187339, year: 2018) and all participants gave written informed consent before the beginning of the experiment. All data were analyzed and reported anonymously.

## Results

Despite the limited sample size, this first set of participants allowed us to validate the experimental procedure, to understand whether the time assigned to each phase was well estimated and to have feedback concerning some interaction features specifically designed for CASTLE. All participants were able to understand how to navigate the scenarios and interact with the different items. The time of each phase appeared sufficient for participants to navigate and interact with the virtual environment.

Below, we first report the distribution of participants’ responses to the questionnaire items for each scenario. These data give a general idea of how each aircraft environment is perceived. Additionally, for each participant we also calculated the median comfort score for each environment, which we used to make a descriptive comparison of the overall level of comfort across the different environments.

### Cabin

**Figure 6:**
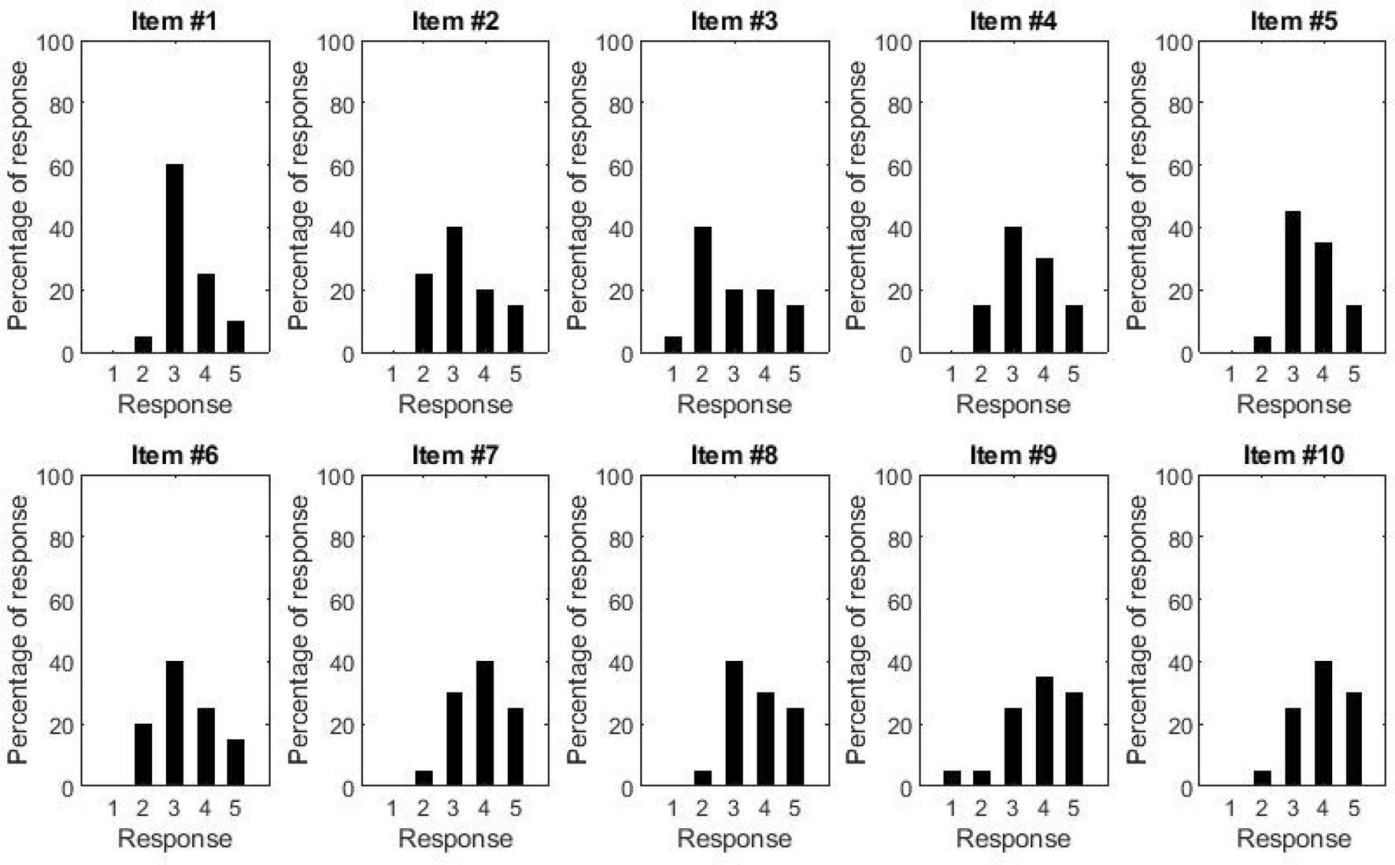
Histograms showing the distribution of responses to the questions about the seat (1–4), the cabin lining (5–6) and the stowage bin (7–10).

No specific issue arose from the virtual navigation of the galley, since all subjects were able to complete the navigation and interact with the seat and stowage bin. Figure 6 shows the distribution of responses to the questions about the seat (items #1–4), the cabin lining (items #5–6) and the stowage bin (items #7–10). Regarding the seat, we find that the majority of participants was pleased with the seat, with a score of 3 or above on all seat items (excluding item #3). Nevertheless, it should be noted that the response that was given most often was “slightly agree”, suggesting that some modifications may be needed to improve the comfort of the seat. In particular, when considering item #3, 45% of participants disagreed that had the feeling of being in a spacious environment. Similarly, regarding the cabin lining (items #5–6), although the majority of participants had a score of 3 or above, the response that was given most often was “slightly agree”. Such distribution of responses may have arisen because the colour and material finishing had not been implemented yet. Finally, regarding the stowage bin (items #7–10), the majority of participants was positive (score 3 or above) about its style and design and about it being spacious enough for the luggage and easy to reach.

### Lavatory

**Figure 7:**
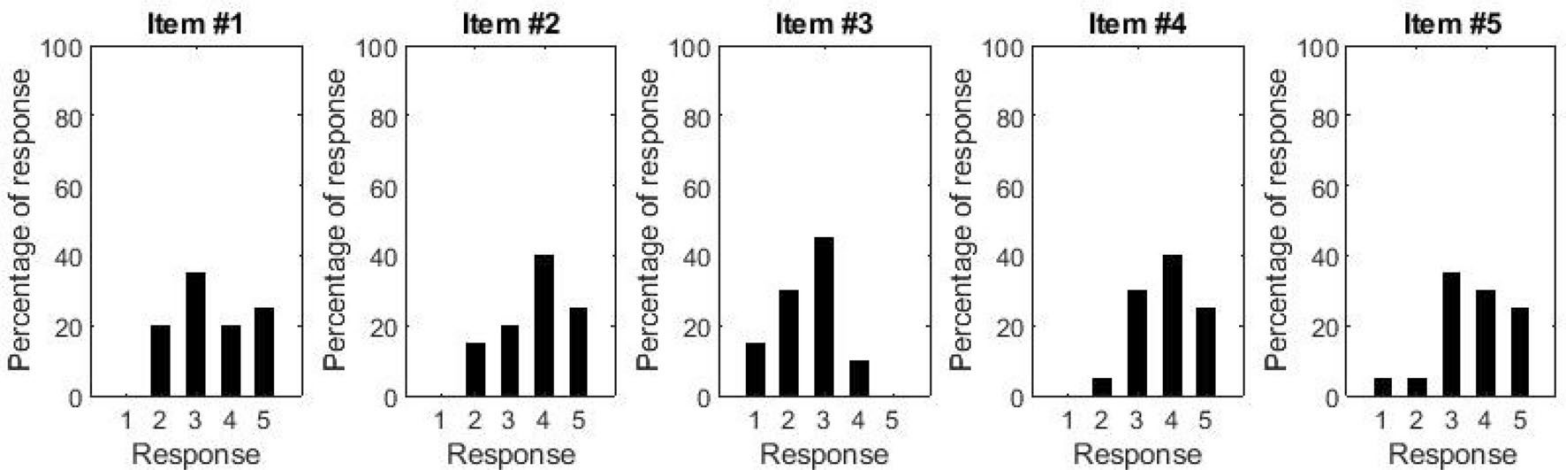
Histograms showing the distribution of responses to the questions about the lavatory.

Figure 7 shows the distribution of responses to the lavatory questionnaire. When considering item #3, no participant strongly agreed that the lavatory appeared spacious and 40% of participants disagreed or strongly disagreed with it, suggesting that some modifications may be needed to improve the size of the lavatory. In contrast, the majority of participants (90%) was pleased with the aesthetics of the lavatory, thought the lavatory seemed comfortable (score of 3 or above on item #2 and item #5 respectively), and 95% thought it appeared easy to use (score of 3 or above on item #4).

### Galley

**Figure 8:**
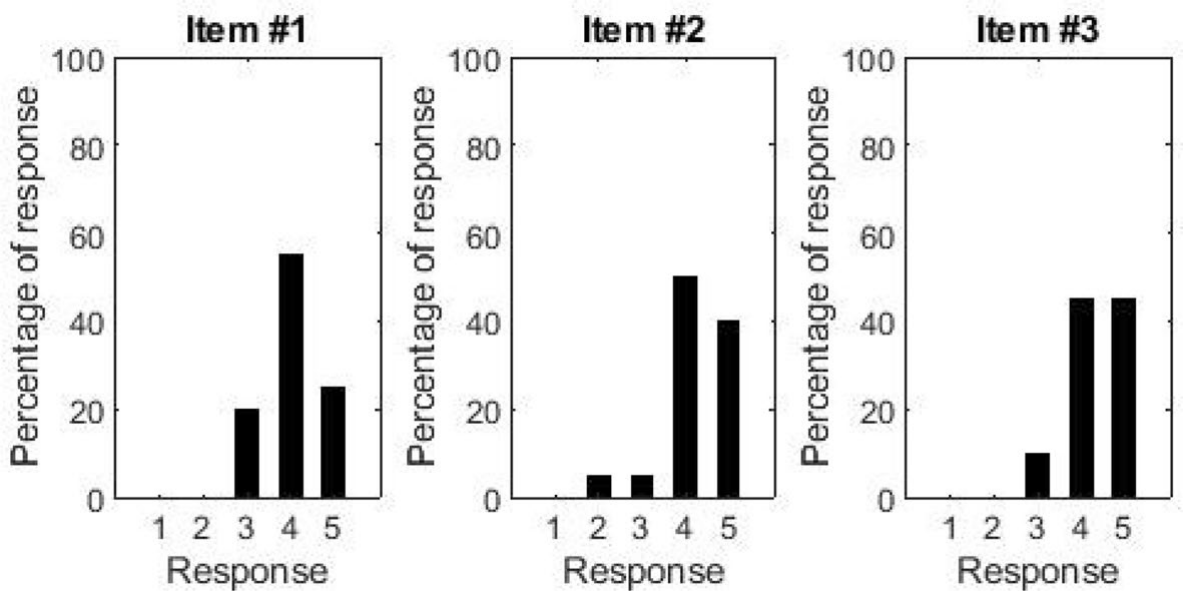
Histograms showing the distribution of responses to the questions about the galley.

Figure 8 shows the distribution of responses to the galley questionnaire. Overall, the distribution of responses appears more homogenous than that for the cabin and lavatory, with no participants strongly disagreeing on any of the items and only 5% of participants mostly disagreeing on item #2. Specifically, all participants were pleased with the style of the galley (score 3 or above on item #1), and in particular 80% of participants mostly or strongly agreed with the statement. Similarly, 90% of participants mostly or strongly agreed that the galley was easy to clean (item #3) and its equipment easy to reach (item #2).

### Overall comfort for each environment

**Figure 9:**
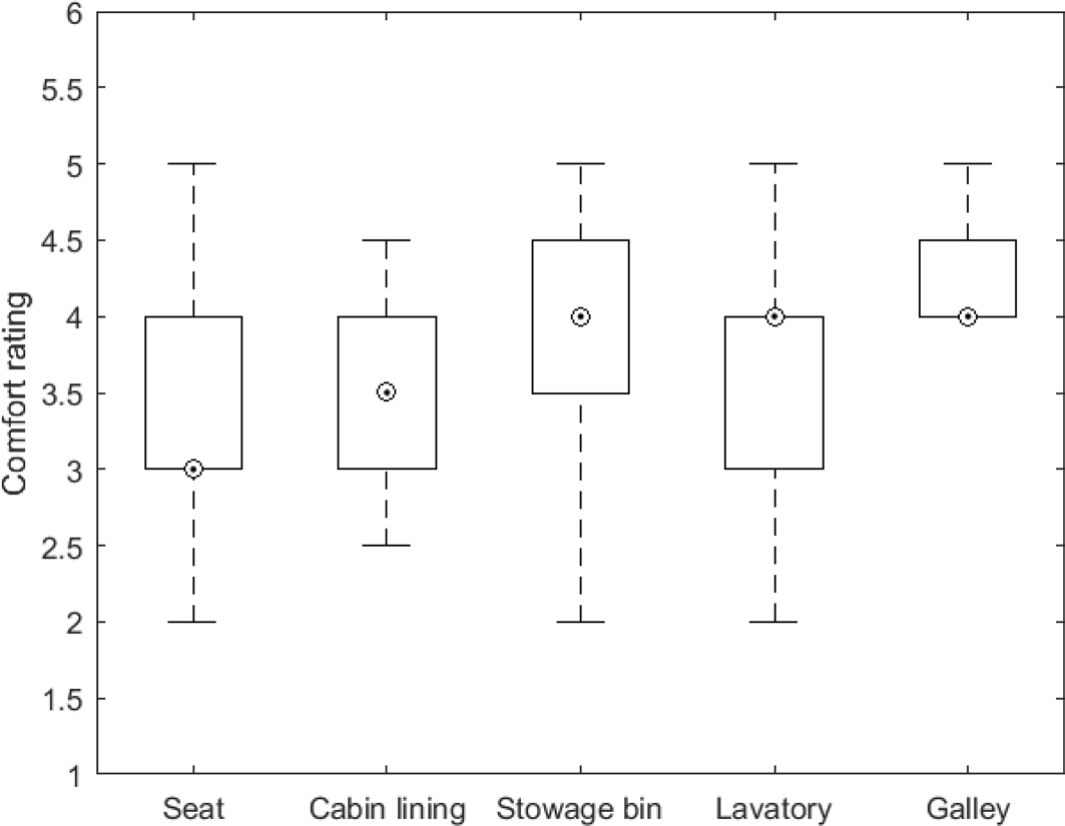
Box plots showing the distribution of participants’ median comfort scores for each environment. On each box, the square within the circle indicates the median, and the bottom and top edges of the box indicate the 25th and 75th percentiles, respectively.

Figure 9 shows the distribution participants’ median comfort scores for each environment. Note that given that the majority of the questions concerned the evaluation of each environment’s space, the overall comfort rating could be dominated by such evaluation. Overall, the seat appears the environment with the lowest median comfort score, although 75% of participants had a median equal to or above 3. The stowage bin, lavatory and galley had the same and highest median comfort score. Despite this, while for the galley all participants scored 4 or above and for the stowage bin 75% scored 3.5 or above, for the lavatory, 75% of participants scored 4 or below.

## Conclusions

In this paper we present the experimental procedure for the Human in the Loop tests in Virtual Reality developed in the framework of the CASTLE project. The project aims to deliver innovative cabin interiors solutions using a holistic approach to evaluate comfort and wellbeing of users. At the early design stage, before the actual manufacturing of items, this evaluation approach is composed of different tools, primary Virtual Manikins software and Virtual Environments. The former allows to perform exact measures for certain human percentiles on the ergonomics performances, while the latter complements it in order to offer a better comprehension of aspects related to the mental processes that lead to the positive or negative impact on the human well being given the actual experience of passengers. Such mental processes have not been modelled yet. Therefore, the scenarios presented in this paper have been designed in order to capture specific aspects, such as, for example, the sense of comfort or discomfort that we feel when we give a first sight to the seat we are approaching or to the space where to load our luggage. In addition, the Virtual Environment has been integrated with interaction features specifically designed for this application.

The work represents the research conducted at this stage of the project that includes the study of the tools and methods, the preparation of the Virtual Reality environments, the CAD processing to gather the correct virtual prototypes, the design of the subjective data collection process and the preliminary tests.

On the one hand, the work is to be regarded as a pilot study since the limited number of participants and the specific category of subjects (students). In fact this is the first run of a wider experimental and iterative campaign under development in the framework of the ongoing project. On the other hand, the data collected represent a first insight in the effectiveness of the experimental procedure. Specifically, all participants were able to successfully complete the scenarios and give a subjective evaluation on the items encountered in each scenario. Therefore, a reproducible methodology for conducting further campaigns and create comparative data is now available.

Taken together, results suggest that the stowage bin and the galley were attributed the highest degree of comfort. In contrast, while the lavatory was perceived as comfortable in terms of its aesthetic, participants expressed less comfort when evaluating it in terms of its space. Also regarding the seat, a certain degree of discomfort was reported when evaluating its space. In the future, changes to the scenarios or to the questions should be made in order to reduce the proportion of this type of response and obtain more conclusive results. In addition, representatives of other classes, such as older people, could be included in further studies.
